# The development of tissue handling skills is sufficient and comparable after training in virtual reality or on a surgical robotic system: a prospective randomized trial

**DOI:** 10.1007/s00464-024-10842-7

**Published:** 2024-04-17

**Authors:** Felix von Bechtolsheim, Andreas Franz, Sofia Schmidt, Alfred Schneider, Felicitas La Rosée, Olga Radulova-Mauersberger, Grit Krause-Jüttler, Anja Hümpel, Sebastian Bodenstedt, Stefanie Speidel, Jürgen Weitz, Marius Distler, Florian Oehme

**Affiliations:** 1 Department of Visceral, Thoracic, and Vascular Surgery, Faculty of Medicine and University Hospital Carl Gustav Carus, TUD Dresden University of Technology, Fetscherstraße 74, 01307 Dresden, Germany; 2 Centre for Tactile Internet with Human-in-the-Loop (CeTI), Technische Universität Dresden, Dresden, Germany; 3 National Center for Tumor Diseases (NCT/UCC), Dresden, Germany; 4 Department of Translational Surgical Oncology, National Center for Tumor Diseases (NCT/UCC Dresden), Dresden, Germany

**Keywords:** Robotic surgery, Training, Tissue handling, Virtual reality, Force

## Abstract

**Background:**

Virtual reality is a frequently chosen method for learning the basics of robotic surgery. However, it is unclear whether tissue handling is adequately trained in VR training compared to training on a real robotic system.

**Methods:**

In this randomized controlled trial, participants were split into two groups for “Fundamentals of Robotic Surgery (FRS)” training on either a DaVinci VR simulator (VR group) or a DaVinci robotic system (Robot group). All participants completed four tasks on the DaVinci robotic system before training (Baseline test), after proficiency in three FRS tasks (Midterm test), and after proficiency in all FRS tasks (Final test). Primary endpoints were forces applied across tests.

**Results:**

This trial included 87 robotic novices, of which 43 and 44 participants received FRS training in VR group and Robot group, respectively. The Baseline test showed no significant differences in force application between the groups indicating a sufficient randomization. In the Midterm and Final test, the force application was not different between groups. Both groups displayed sufficient learning curves with significant improvement of force application. However, the Robot group needed significantly less repetitions in the three FRS tasks Ring tower (Robot: 2.48 vs. VR: 5.45; *p* < 0.001), Knot Tying (Robot: 5.34 vs. VR: 8.13; *p* = 0.006), and Vessel Energy Dissection (Robot: 2 vs. VR: 2.38; *p* = 0.001) until reaching proficiency.

**Conclusion:**

Robotic tissue handling skills improve significantly and comparably after both VR training and training on a real robotic system, but training on a VR simulator might be less efficient.

**Supplementary Information:**

The online version contains supplementary material available at 10.1007/s00464-024-10842-7.

Robot-assisted surgery (RAS) has become an accepted and standardized surgical approach following the rapid development of technology and scientific knowledge over the last two decades [1, 2]. Laparoscopy is still the method of choice, but the proportion of robotic surgery in minimally invasive abdominal surgery continues to increase [3].

Still, whereas laparoscopic training has been widely implemented in surgical residency and is sometimes even required for board certification as a surgeon, robotic training does not have nearly the same status in surgical residency and education [4, 5].

As a consequence, over one-third of surgical trainees in the USA felt inadequately trained in RAS. Similarly, in the UK and Ireland, the percentages of trainees with subjectively inadequate training were 86.2% and 91.7%, respectively [6]. At the same time, sufficient training is required because RAS is highly demanding owing to the complex technology, elaborate handling, and limitations, such as limited field of view and lack of haptic feedback [7]. In particular, the latter remains a major challenge for surgeons because most modern robotic surgery systems provide no or only rudimentary haptic feedback. However, experienced surgeons are able to compensate for this lack of haptic feedback compared to less experienced surgeons, ultimately resulting in less force being applied to the tissue [8]. It is speculated to be primarily based on a visual assessment of instrument–tissue interactions [9].

Although investigation of the force interaction between surgical instruments and tissue is a relatively new field of surgical research, it has been established that correct tissue handling is essential for surgical performance and patient safety [10]. Tissue handling as such is only part of a surgeon's technical skills, but these technical skills are generally associated with fewer complications, a lower risk of bleeding or the risk of re-operation [11]. Also, for laparoscopic surgery Tang et al. could demonstrate a correlation between excessive force and surgical errors [12, 13]. A similar relationship can be assumed in RAS. However, it is still unknown how the established basic skills training curricula for RAS affect tissue handling skills. Consequently, this trial aimed to investigate the learning curves and potential differences in tissue handling skills depending on the training modality (virtual reality training vs. training on a robotic system) after a basic robotic skills curriculum.

## Methods

This investigation was designed and conducted as a prospective, randomized, controlled study. The study protocol was approved by the ethics committee of TU Dresden (decision number EK 285072016) and registered at the German Clinical Trials Register (Registry number DRKS00033919). All experimental methods were performed in accordance with the relevant guidelines, and this article was drafted and written in accordance with the CONSORT statement [14].

### Participants

Written informed consent was obtained from all participants. Participants were medical students and first-year surgical residents without any formal experience in robotic surgery. Previous experience in robotic surgery or participation in RAS training courses was exclusion criteria. A questionnaire asking about basic participant characteristics (age, surgical and robotic experience, etc.) was completed by all participants.

### Study design

**Fig. 1:**
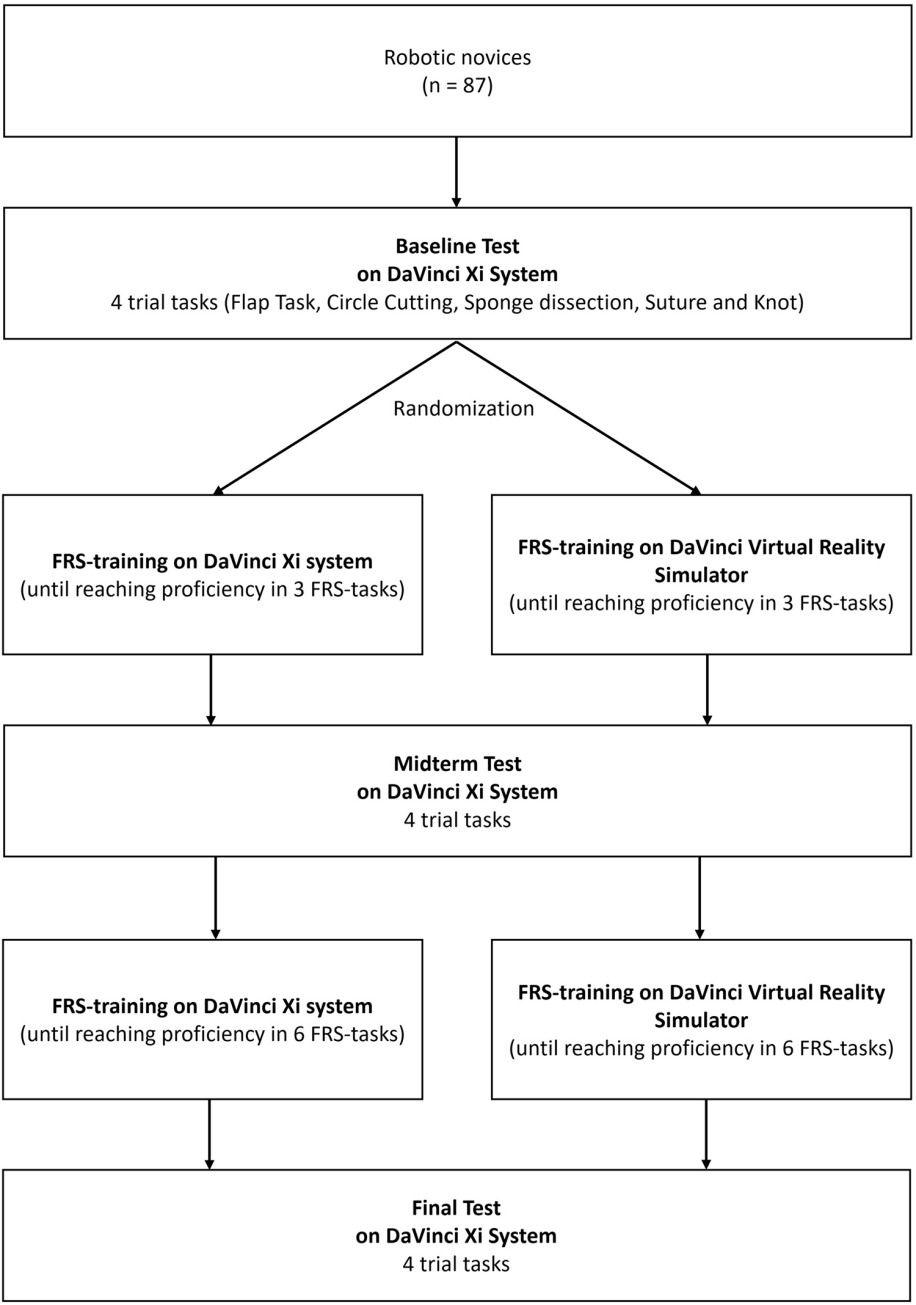
Trial scheme

All participants received an individual introduction into the surgeon’s console of the DaVinci Xi® surgical system (Intuitive Surgical, Sunnyvale, CA, USA). Subsequently, all participants had to perform four trial tasks as Baseline test on the DaVinci Xi® surgical system (Fig. 1). The force interactions of the robotic instruments were measured during the trial tasks using a force-measuring device. Based on their respective performance in the Baseline test, the investigators randomized the participants block-wise (block-size six to 10) into two groups to ensure comparable baseline performance between the groups. Each group received the same training curriculum in two training sessions either on the DaVinci Xi virtual reality simulator (VR group) or on the real DaVinci Xi surgical system (Robot group). The trial tasks were repeated as Midterm test between the training sessions and as Final test after both training sessions on the DaVinci Xi® surgical system, respectively. Tests and training sessions were conducted on separate days and always within one week.

### Training pathway

Both the VR group and Robot group underwent the same Fundamentals of Robotic Surgery (FRS) training curriculum on their respective training modality. Participants had to perform six FRS tasks in two training sessions of individual length (1st training session: Ring Tower Transfer, 4th Arm Cutting, and Railroad Track and 2nd training session: Knot Tying, Puzzle Piece Dissection, and Vessel Energy Dissection) until reaching a certain level of proficiency (min. 90 points) for each task twice. The proficiency score was calculated for both groups using the same modified scoring algorithm based on the SIMSCORE Version: BETA of the DaVinci Xi® Virtual Reality Simulator. This scoring algorithm considers task time and predefined errors as defined by SIMSCORE Version: BETA. However, the economy of motion was not integrated because it could not be measured for the Robot group.

### Trial tasks

The four trial tasks for Baseline, Midterm, and Final test were the Flap task, the Precise Cut task, the Dissection task, and the Suture and Knot task (Supplementary Material). These trial tasks were designed or selected to require surgical skills similar to those of the FRS tasks. The deviation from FRS tasks ensured that neither of both groups had any advantage in performing the trial tasks.

### Endpoints

The primary endpoint was tissue handling, represented by the mean non-zero force [N] and the peak force [N] exerted during the trial tasks in the Baseline, Midterm, and Final tests [15, 16]. The force parameters were measured using a force-measuring device (ForceTrap®, MediShield B.V., Delft, The Netherlands). This device can measure the force interaction in three dimensions (Unit: Newton) between robotic instruments and the respective tasks mounted on a platform attached to the ForceTrap (Supplementary Material) [17].

The force inputs were analyzed using the ForceSense software (MediShield B.V., Delft, The Netherlands) whose parameters are defined as follows:

- Mean non-zero force: the mean of all forces during a task excluding all periods with zero force exertion
- Peak force: maximum force applied during a task

The secondary endpoints were the time of completion [s] and the occurrence of predefined errors during any trial task (Supplementary Material). The learning curves were defined as the change in task time, mean non-zero force, and peak force over the course of the trial tasks (baseline, midterm, and final). For the FRS training tasks, the number of repetitions until attaining proficiency at the respective modality was counted.

### Statistical analysis

All the statistical analyses were performed using SPSS version 26 (IBM Corp., Armonk, NY, USA). Kolmogorov–Smirnov tests were used to check the normality of the continuous data. Variables are represented either as mean values (mean) and standard deviations (SD) for continuous variables or as distributions of frequencies. Differences between groups were tested using the Student’s *t*-test or Mann–Whitney *U*-Test depending on the distribution of normality. For the analysis of varying error incidence, a Chi^2^-test was chosen. Learning curves were analyzed using a general linear model with repeated measurements and post-hoc Bonferroni corrections. The threshold for the level of significance was set at *p* = 0.05. A preceding power analysis was not performed owing to the lack of data on the potential effect size.

## Results

### Basic participant characteristics

For this trial, 87 participants were randomized into a Robot group (*n* = 44) and into a VR group (*n* = 43). The 50 (57.8%) female participants outweighed the 37 (42.5%) male participants. The mean age was 24.9 (SD 3.5) years. None of the participants had attended a training course on robot-assisted surgeries. However, 35 participants (40.2%) reported previous experience in laparoscopic surgery (e.g., assistance, camera holding), 28 (32.2%) participants completed a training course for laparoscopic surgery, and 9 (10.3%) had used the Da Vinci Surgical System before but did not receive any formal training or participated in robotic surgeries as console surgeons (Table 1).

**Table 1:** Basic participant characteristics

	*n* (%)	Mean (SD)
Age [years]		24.9 (3.5)
Sex		
Male	37 (42.5)	
Female	50 (57.8)	
Participation in a laparoscopic training course	28 (32.2)	
Practical experience in laparoscopic surgery	35 (40.2)	
Practical experience with the DaVinci surgical system	9 (10.3)	
1–2 h	7 (8)	
3–5 h	2 (2.3)	

### Comparison between the Robot and VR group: Baseline test

There were no significant differences between the Robot and VR groups in any task in the Baseline test for completion time, mean non-zero force, or peak force (Table 2). In addition, both groups made comparable numbers of major errors during the Baseline test (Table 3).

**Table 2:** Comparison of completion time and force values between Robot and VR group

		Baseline test			Midterm test			Final test		
		Robot group (*n* = 43)	VR group (*n* = 44)		Robot group (*n* = 42)	VR group (*n* = 41)		Robot group (*n* = 40)	VR group (*n* = 38)	
		Mean (SD)	Mean (SD)	*p*-Value	Mean (SD)	Mean (SD)	*p*-Value	Mean (SD)	Mean (SD)	*p*-Value
Flap	Time [s]	101.7 (49.1)	103.9 (53.4)	0.741	59.2 (15.2)	73.4 (24.1)	**0.007**	59 (20.6)	64.2 (19.5)	0.266
	Mean force [N]	1 (0.7)	1 (0.5)	0.812	0.7 (0.3)	0.9 (1.1)	0.981	0.8 (0.9)	0.9 (0.9)	0.815
	Peak force [N]	4.8 (3.6)	4.9 (3.3)	0.839	2.6 (2.3)	2.7 (2.8)	0.917	2.4 (2.9)	3.2 (2.7)	0.113
Precise cut	Time [s]	274.3 (80.6)	314.2 (138.1)	0.248	196.4 (70.5)	205.7 (71.8)	0.616	160.9 (51.6)	175.6 (61.1)	0.255
	Mean force [N]	0.7 (0.6)	0.8 (0.4)	0.144	0.6 (0.3)	0.7 (0.5)	0.339	0.5 (0.3)	0.5 (0.3)	0.826
	Peak force [N]	3.2 (3.3)	3.8 (3.4)	0.259	2.1 (1.9)	2.7 (2.3)	0.127	1.8 (1.9)	1.9 (1.4)	0.115
Dissection	Time [s]	563.6 (162.1)	573.6 (214.3)	0.855	411.1 (99.6)	421.7 (122.7)	0.667	399.6 (131.5)	401 (138.6)	0.964
	Mean force [N]	1.4 (0.5)	1.5 (0.6)	0.248	1.2 (0.5)	1.2 (0.4)	0.68	1.2 (0.5)	1.3 (0.4)	0.206
	Peak force [N]	5.5 (2)	5.8 (2.9)	0.959	4.4 (1.7)	4.4 (1.4)	0.915	4.5 (2.1)	4.4 (1.3)	0.519
Suture and knot	Time [s]	344 (152.1)	333.7 (113.6)	0.941	183.8 (72.1)	227.2 (68.4)	**0.006**	170.5 (58.9)	182.4 (60.9)	0.384
	Mean force [N]	1.7 (0.5)	1.8 (0.5)	0.401	1.5 (0.4)	1.5 (0.4)	0.857	1.2 (0.4)	1.3 (0.4)	0.103
	Peak force [N]	6.6 (2.8)	6.9 (3)	0.405	5.2 (2.1)	5.2 (1.9)	0.921	4.1 (2)	4.7 (2.2)	0.086

Significant *p*-values marked in bold

**Table 3:** Comparison of error occurrence between Robot and VR group

		Baseline test			Midterm test			Final test		
		Robot group (*n* = 43)	VR group (*n* = 44)		Robot group (*n* = 42)	VR group (*n* = 41)		Robot group (*n* = 40)	VR group (*n* = 38)	
		*n* (%)	*n* (%)	*p*-Value	*n* (%)	*n* (%)	*p*-Value	*n* (%)	*n* (%)	*p*-Value
Flap	General errors	3 (6.8)	3 (7.0)	0.977	2 (4.5)	2 (4.7)	0.887	0 (0)	2 (4.7)	0.256
Precise cut	General errors	14 (31.8)	9 (20.9)	0.25	6 (13.6)	9 (20.9)	0.559	0 (0)	2 (4.7)	0.256
Dissection	General errors	22 (50)	26 (60.5)	0.418	10 (22.7)	9 (20.9)	0.879	8 (18.2)	3 (7.0)	0.286
Suture and knot	General errors	20 (45.5)	23 (53.5)	0.454	8 (18.2)	7 (16.3)	0.874	1 (2.3)	2 (4.7)	0.765
	Suture tightness error	12 (27.3)	9 (20.9)	0.489	9 (20.5)	13 (30.2)	0.557	12 (27.3)	9 (20.9)	0.769
	Suture precision error	8 (18.2)	6 (14.0)	0.592	1 (2.3)	4 (9.3)	0.366	0 (0)	4 (9.3)	0.101

Significant *p*-values marked in bold

### Comparison between the Robot and VR group: Midterm test

There was a slight loss to follow-up in the Midterm test with 42 and 41 participants remaining in the Robot and VR group, respectively. In the Midterm test, the Robot group showed significantly faster task completion times in Flap task (Robot:59.2 s vs. VR:73.4 s; *p* = 0.007) and Suture and Knot task (Robot:183.8 s vs. VR:227.2 s; *p* = 0.006) (Table 2). Except for the mean non-zero force in the Dissection and the Suture and Knot task, all other results were more favorable in the Robot group compared to the VR group. However, these differences were not statistically significant. Similarly, on the Midterm test, the number of errors did not differ between the two groups for any task (Table 3).

### Comparison between the Robot and VR group: Final test

Despite not reaching significance, the Final test showed a clear tendency in favor of the Robot group (Table 2). In the Final test, a further slight loss to follow-up with 40 and 38 participants in the Robot and VR group, respectively, was noted. Apart from a better peak-force values by the VR group in the Dissection task, the Robot group performed better in the Flap task (Robot:2.4 N vs. VR:3.2 N; *p* = 0.113), the Precise Cut task (Robot:1.8 vs. VR:1.9; *p* = 0.115), and in the Suture and Knot task (Robot:4.1 N vs. VR:4.7 N; p = 0.086). The differences in the mean non-zero force were less pronounced, but again in favor of the Robot group in the Flap task (Robot:0.8 N vs. VR:0.9 N; *p* = 0.815), the Dissection task (Robot:1.2 N vs. VR:1.3 N; *p* = 0.206), and the Suture and Knot task (Robot:1.2 N vs. VR:1.3 N; *p* = 0.103). In the Precise Cut task, both groups performed equally in terms of the mean non-zero force (Robot:0.5 N vs. VR:0.5 N; *p* = 0.826).

The tendency in favor of the Robot group could also be seen regarding the task times for Flap task (Robot:59 s vs. VR:64.2 s; *p* = 0.266), Precise Cut task (Robot:160.9 s vs. VR:175.6 s; *p* = 0.255), Dissection task (Robot:399.6 s vs. VR:401 s; *p* = 0.964), and Suture and Knot task (Robot:170.5 s vs. VR:182.4 s; *p* = 0.384).

There were no significant differences in the occurrence of errors and no tendencies were observed in the Final test (Table 3).

### Learning curve for task time

**Fig. 2:**
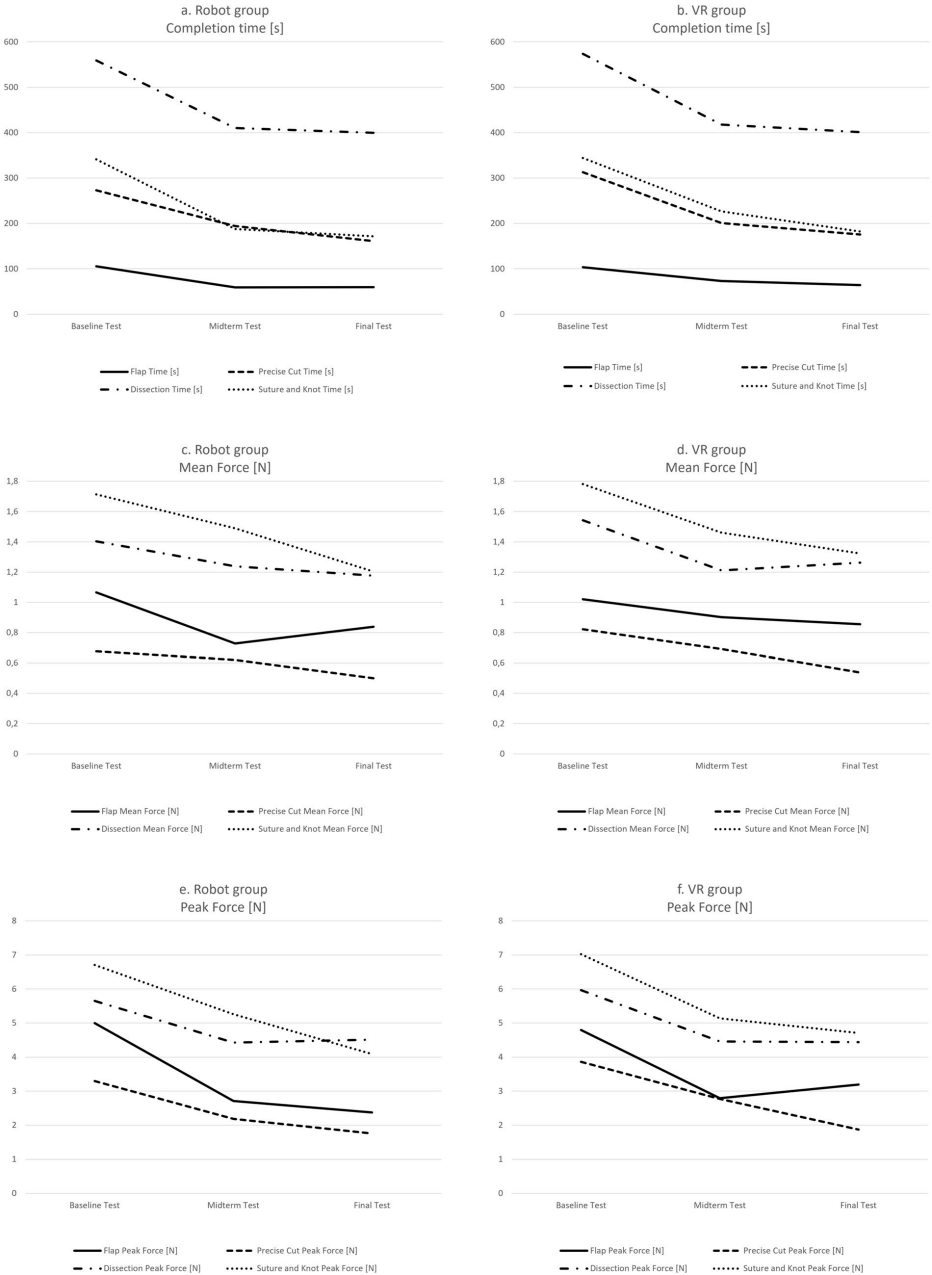
**a**–**f** Learning curves for completion time, mean non-zero force, and peak force for each task

Both groups showed an overall significant improvement in task completion time for all tasks (Table 4; Fig. 2a). However, the Robot group improved significantly mainly between Baseline and Midterm test in the Flap task (*p* < 0.05), the Dissection task (*p* < 0.05), and the Suture and Knot task (*p* < 0.05) and the further development between Midterm and Final test did not show significant differences. A significant improvement between all tests was observed only in the Precise Cut task for the Robot group.

**Table 4:** ANOVA of completion time, mean and peak force per task

		Robot group				VR group			
		Baseline test	Midterm test	Final test		Baseline test	Midterm test	Final test	
		Mean (SD)	Mean (SD)	Mean (SD)	*p*-Value	Mean (SD)	Mean (SD)	Mean (SD)	*p*-Value
Flap	Time [s]	105.61 (8.62)	59.26 (2.48)	59.69 (3.43)	** < 0.05**^**a**^	103.67 (9.16)	73.03 (4.06)	64.16 (3.17)	** < 0.05**
	Mean force [N]	1.07 (0.12)	0.73 (0.06)	0.84 (0.15)	> 0.05	1.02 (0.09)	0.9 (0.19)	0.86 (0.15)	> 0.05
	Peak force [N]	5 (0.6)	2.71 (0.4)	2.38 (0.49)	** < 0.01**^**b**^	4.79 (0.53)	2.79 (0.47)	3.2 (0.44)	** < 0.05**^**b**^
Precise cut	Time [s]	273.18 (12.94)	194.27 (11.18)	160.89 (8.16)	** < 0.001**	313.05 (23.25)	200.68 (11.1)	175.56 (9.91)	** < 0.05**
	Mean force [N]	0.68 (0.1)	0.62 (0.06)	0.5 (0.04)	> 0.05	0.82 (0.07)	0.69 (0.08)	0.54 (0.04)	** < 0.01**^**c**^
	Peak force [N]	3.29 (0.55)	2.19 (0.3)	1.76 (0.3)	** < 0.01**^**c**^	3.86 (0.58)	2.77 (0.39)	1.87 (0.23)	** < 0.01**^**c**^
Dissection	Time [s]	559.17 (26.32)	410.35 (15.43)	399.63 (20.8)	** < 0.05**^**a**^	573.96 (35.03)	417.84 (20.1)	401.02 (22.49)	** < 0.05**^**a**^
	Mean force [N]	1.4 (0.09)	1.24 (0.08)	1.18 (0.08)	> 0.05	1.54 (0.11)	1.21 (0.07)	1.26 (0.07)	** < 0.05**^**b**^
	Peak force [N]	5.65 (0.31)	4.43 (0.28)	4.51 (0.34)	** < 0.05**^**b**^	5.96 (0.49)	4.46 (0.23)	4.44 (0.21)	** < 0.05**^**b**^
Suture and knot	Time [s]	341.24 (25.01)	187.97 (11.63)	171.58 (9.5)	** < 0.05**^**a**^	343.98 (18.54)	226.7 (11.18)	182.43 (9.88)	** < 0.001**
	Mean force [N]	1.71 (0.08)	1.49 (0.07)	1.2 (0.07)	** < 0.01**^**d**^	1.78 (0.08)	1.46 (0.06)	1.32 (0.07)	** < 0.01**^**b**^
	Peak force [N]	6.71 (0.45)	5.26 (0.35)	4.09 (0.32)	** < 0.05**^**b**^	7.02 (0.49)	5.14 (0.29)	4.71 (0.35)	** < 0.01**^**b**^

Significant *p*-values marked in bold

^a^Significant *p*-values only between test trial 1 vs. 2 and 1 vs. 3

^b^Significant *p*-values only between test trial 1 vs. 2 and 1 vs. 3

^c^Significant *p*-values only between test trial 1 vs. 3

^d^Significant *p*-values only between test trial 1 vs. 3 and 2 vs. 3

For the VR group, the improvement of task completion time was significant between all tests in the Flap task (*p* < 0.05), the Precise Cut task (*p* < 0.05), and the Suture and Knot task (*p* < 0.001) (Table 4; Fig. 2b). In the Dissection task, significant improvement was observed only between Baseline and Midterm (*p* < 0.05) but not between the Midterm and Final tests.

### Learning curve for mean non-zero force

Regarding the mean non-zero force, the Robot group showed a tendency but no significant improvement in the Flap task, Precise Cut task, or Dissection task (Table 4; Fig. 2c). An overall improvement with the steepest improvement between Midterm and Final test could be seen in the Suture and Knot task (*p* < 0.01).

In the VR group, the learning curve for mean non-zero force was not significant for the Flap task (*p* > 0.05) (Table 4; Fig. 2d). The Precise Cut task showed an significant overall improvement (*p* < 0.01). Here, an overall improvement with the steepest improvement between Baseline and Midterm test was observed in the Dissection task (*p* < 0.05) and Suture and Knot task (*p* < 0.01).

### Learning curve for peak force

The learning curves of peak-force exertion showed a significant and comparable improvement in both groups for all tasks (Table 4; Fig. 2e). For the Robot group, a significant overall improvement of peak forces was seen in all tasks (Flap: *p* < 0.01; Precise Cut: *p* < 0.01; Dissection: *p* < 0.05; Suture and Knot: *p* < 0.05). With the exception of the Precise Cut task, all other tasks additionally showed a significant improvement between Baseline and Midterm test.

The VR group (Table 4; Fig. 2f) showed a significant overall improvement in peak forces (Flap: *p* < 0.05; Precise Cut: *p* < 0.01; Dissection: *p* < 0.05; Suture and Knot: *p* < 0.01). Again, with the exception of the Precise Cut task, all other tasks also improved significantly between Baseline and Midterm test.

### Learning efficiency

The number of FRS task repetitions (Ring Tower Transfer, 4th Arm Cutting, Railroad Track, Knot Tying, Puzzle Piece Dissection, Vessel Energy Dissection) performed by each participant until reaching proficiency was counted and compared between the two groups (Table 5). Interestingly, the VR group needed significantly more repetitions in the FRS Ring tower (Robot:2.48 vs. VR: 5.45; *p* < 0.001), Knot Tying (Robot: 5.34 vs. VR: 8.13; *p* = 0.006), and Vessel Energy Dissection (Robot: 2 vs. VR: 2.38; *p* = 0.001) tasks until reaching proficiency.

**Table 5:** Number of sessions for each training task until reaching sufficiency

	Robot group	VR group	*p*-Value
	Mean (SD)	Mean (SD)	
Ring tower transfer [*n*]	2.5 (0.8)	5.5 (2.7)	** < .001**
4th arm cutting [*n*]	2.6 (0.7)	2.9 (1.1)	0.18
Railroad track [*n*]	4.5 (1.9)	5.4 (2.2)	0.07
Knot tying [*n*]	5.3 (3.1)	8.1 (5)	**0.006**
Puzzle piece dissection [*n*]	5.5 (1.7)	4.5 (1.8)	0.22
Vessel energy dissection [*n*]	2 (0)	2.4 (1)	**0.001**

significant *p*-values marked in bold

## Discussion

Recently, various training curricula for robot-assisted surgery have been developed, tested, and partially validated. FRS have been developed for learning basic robotic surgery skills and are available on most VR training simulators, as well as training on a real-world robotic surgical system.

Regarding task completion time and occurrence of errors, VR training has already proven to be as effective as the same curriculum using a robotic surgical system [18]. However, this trial did not include any analysis of tissue handling or objective force measurement, although it is well known that the lack of haptic feedback remains a major shortcoming of RAS. Hence, data regarding the force interaction between robotic instruments and tissues are scarce, and consequently, tissue handling has never been the focus of basic skills training curricula for RAS.

The present trial provided detailed information on the learning curve for tissue handling over an FRS-based course for robotic novices. Both training on the robotic surgical system and using the VR simulator showed a significant reduction in tissue handling forces. In general, both groups showed steep and significant improvements in time and force exertion over the course of the training. This indicates that the FRS curriculum-driven training can sufficiently help to develop tissue handling skills, even though the FRS was not specifically designed for teaching and rating tissue handling.

Our findings are supported by a recent study that demonstrated similar reductions in force and time for a repeated robotic suturing task, but without comparison with VR training. In addition, the range of the peak and mean non-zero forces observed by Rahimi et al. was comparable to our measurements [16].

The application of force is significantly influenced by the tissue type, haptic feedback combined with visual assessment, and surgical experience [19]. It is therefore not surprising that there are significant differences between surgical experts and beginners, with the latter using more excessive force [20, 21]. However, these studies did not take RAS into account, whereby in RAS the visual assessment of the interaction between robotic instruments and tissue is thought to play the major role in compensating for missing haptic feedback [9, 22]. Unlike surgical experience, such visual assessment can be trained, but requires a realistic training scenario with high-quality simulation of force interaction between instruments and tissue. It was questionable if VR simulator can provide such a realistic force and haptic feedback simulation [23]. Overtoom et al. were the first to conclude that laparoscopic VR training with simulated forces and haptic feedback leads to only minor improvements for surgical novices compared to force feedback provided by a laparoscopic box-trainer [24].

For RAS, a similar comparison has been missing thus far, in part because there is no haptic feedback in RAS and therefore the difference between training in VR and on a robotic system may have been considered less relevant; therefore, we aimed to investigate the potential differences in the learning of tissue handling depending on the training modality. Consequently, our data suggest that there are no significant differences in the development of tissue handling skills and force interactions depending on the training modality. The comparable Baseline test showed sufficient randomization into the VR and Robot groups. The Midterm and Final tests showed no significant differences in force exertion between the groups but showed an overall tendency in favor of the Robot group. This indicates a slight advantage of training with a real robotic system.

When comparing the efficiency of VR training with training on a surgical robotic system, we found that the Robot group required significantly fewer sessions for three of the six FRS training tasks (Ring tower, Knot, and Vessel Dissection task) to achieve the required competency. In particular, for the Knot task, the VR group struggled regularly with the simulated thread. Here, the limited realism of the VR simulation could be the cause of the poorer performance and thus lead to a less efficient and even frustrating training experience.

### Strengths and limitations

To the best of our knowledge, this is the first randomized trial to analyze and compare the learning curves of tissue handling between training for RAS using VR simulators and a robotic surgical system. To increase comparability, the participants were purposefully chosen to be naïve to RAS. In particular, the distinction in surgical experience based on the respective tissue handling skills in RAS has been observed previously [16].

The relevance of the observed tissue handling skills and their transferability to the OR remain vague and partially unknown. A force interaction of approximately 2 N is discussed as a threshold for damage to certain tissues (e.g., large intestine) in a suture-like scenario [25]. In our study, both groups exceeded this threshold by far in the Suture and Knot task even after completing the FRS training. Organ-specific differences were shown by the damage occurring after 20 N grasping force in intestine and 1 N grasping force in liver [26, 27]. However, the data supporting these claims were collected in animal experiments and may not be applicable to humans.

The data shown were collected using the most common robotic surgical system, the DaVinci Xi surgical system, and the most common robotic VR trainer, the DaVinci Xi Virtual Reality simulator. However, other robotic systems or VR simulators could potentially yield different results. Novel developments in RAS already provide some sort of haptic feedback and may change the relevance of our findings and conclusions. Alternatively, successful attempts have been made to use optical data to estimate the force estimation [28–31].

Still, the importance of haptic feedback should not be underestimated, since providing haptic and tactile feedback improves the surgeon’s force application toward the tissue, decreasing tissue damage and surgical performance in general [8, 32]. Notwithstanding, the integration of haptic feedback does not exempt the need for training. Singapore et al. concluded that at least for laparoscopy, receiving haptic feedback per se and processing force cues would also require training [23]. Also, future robotic systems might even incorporate feedback technology that exceeds the human tactile sense. It therefore remains to be seen whether and how the implementation of haptic feedback in surgical robotic systems might change RAS training.

Eventually, the exploratory nature of this study meant that a preceding power analysis was not possible. It is therefore possible that this study was underpowered and possible measurement differences would only have become apparent after the inclusion of additional participants.

## Conclusion

Tissue handling in RAS is particularly demanding because of the lack of haptic feedback and requires extensive and specialized training. The results of our study indicate that the FRS curriculum can significantly improve tissue handling, with no differences in outcomes between robotic and VR training modalities. However, VR training might be slightly less efficient in terms of task repetitions needed to reach proficiency.

Still, objective assessment of tissue handling should be integrated into RAS training. Robotic surgical systems with implemented haptic feedback may help improve force exertion and also training for RAS to some extent. However, the transferability of robotic surgery tissue handling skills to the operating room is still unknown and should be the subject of future studies.

### Supplementary Information

Below is the link to the electronic supplementary material.

Supplementary file1 (DOCX 297 kb)

Supplementary file2 (DOCX 14 kb)

Supplementary file3 (DOCX 4163 kb)
