# Supplementary material for: The development of tissue handling skills is sufficient and comparable after training in virtual reality or on a surgical robotic system: a prospective randomized trial
Source: Surg Endosc. 2024 Apr 17;38(5):2900–10. doi: 10.1007/s00464-024-10842-7 (PMC11078795; doi:10.1007/s00464-024-10842-7)
Supplement: Supplementary file 1 — Supplementary file1 (DOCX 297 kb) [file 464_2024_10842_MOESM1_ESM.docx]

**Supplementary Material:**

| **Trial task** | **Description** | **Figure** |
| --- | --- | --- |
| **Flap** | In the flap task (MediShield B.V., Delft, Netherlands), the participants used two forceps to lift a silicone flap attached to one side of the measuring platform. A string was then inserted through the two holes in the silicone flap. | 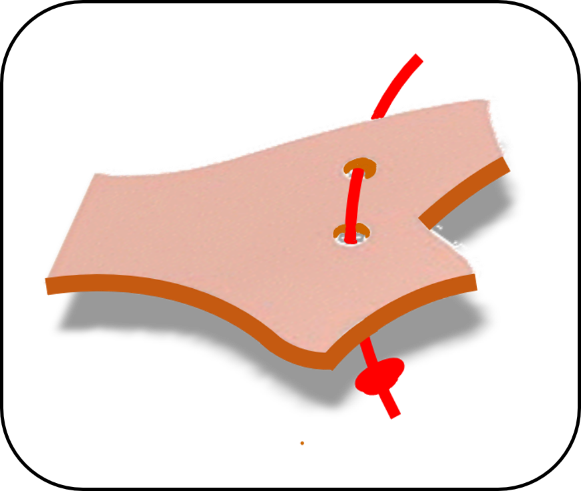 |
| **Precise Cut** | A surgical pad was attached to the measuring platform on four sides. With one set of forceps and scissors, a circle drawn on the pad had to be cut out exactly on the drawn line. | 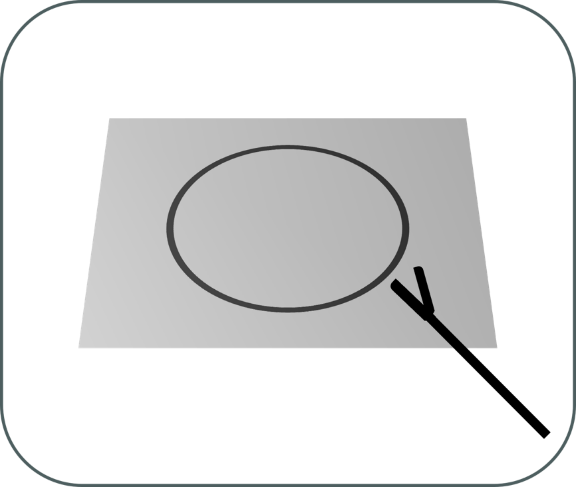 |
| **Dissection** | A sponge with two colored layers was attached to the measurement platform. The participants had to dissect exactly between the two layers using two forceps and scissors. | 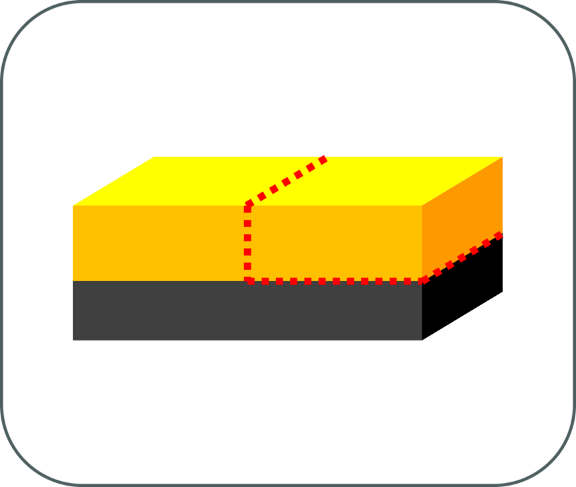 |
| **Suture and Knot** | A penrose drain (Limbs & Things Inc., Savannah, GA, USA) with two dots on each side was attached to the measuring platform. Using a needle holder, a forceps and a 12 cm long Vicryl® 3-0 suture (Ethicon, Raritan NJ, USA), participants had to stitch precisely through both holes and then perform a surgical knot. | 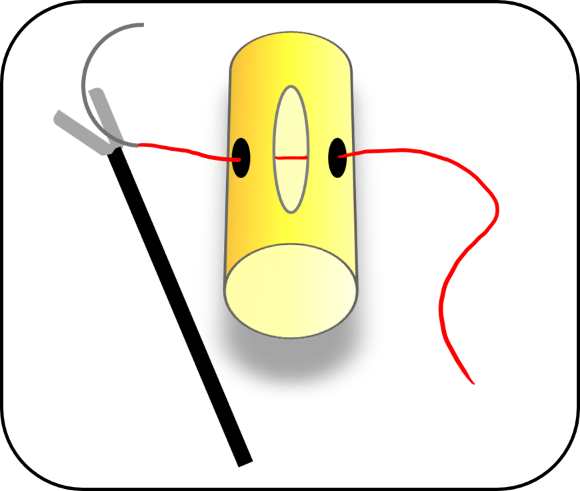 |

Supplementary Material Table 1: Definition and graphic display of each trial tasks
