# Supplementary material for: The development of tissue handling skills is sufficient and comparable after training in virtual reality or on a surgical robotic system: a prospective randomized trial
Source: Surg Endosc. 2024 Apr 17;38(5):2900–10. doi: 10.1007/s00464-024-10842-7 (PMC11078795; doi:10.1007/s00464-024-10842-7)
Supplement: Supplementary file 2 — Supplementary file2 (DOCX 14 kb) [file 464_2024_10842_MOESM2_ESM.docx]

**Supplementary Material:**

|  |  | **Error Definition** |
| --- | --- | --- |
| **Flap** |  | Droping the string; Pulling the string knot through one/both holes; Instrument collision; Instruments out of view |
| **Precise Cut** |  | Cutting > 5mm outside the marked line; Instrument collision; Instruments out of view |
| **Dissection** |  | Tearing of the sponge; Cutting > 5mm outside the marked line; Instrument collision; Instruments out of view |
| **Suture and Knot** | General | Tearing the suture; Instrument collision; Instruments out of view |
|  | Tightness | Loose knot |
|  | Precision | Missing one/both marked dots |

Supplementary Material Table 2: Definition and graphic display of each trial tasks
