# Supplementary material for: The development of tissue handling skills is sufficient and comparable after training in virtual reality or on a surgical robotic system: a prospective randomized trial
Source: Surg Endosc. 2024 Apr 17;38(5):2900–10. doi: 10.1007/s00464-024-10842-7 (PMC11078795; doi:10.1007/s00464-024-10842-7)
Supplement: Supplementary file 3 — Supplementary file3 (DOCX 4163 kb) [file 464_2024_10842_MOESM3_ESM.docx]

**Supplementary Material:**


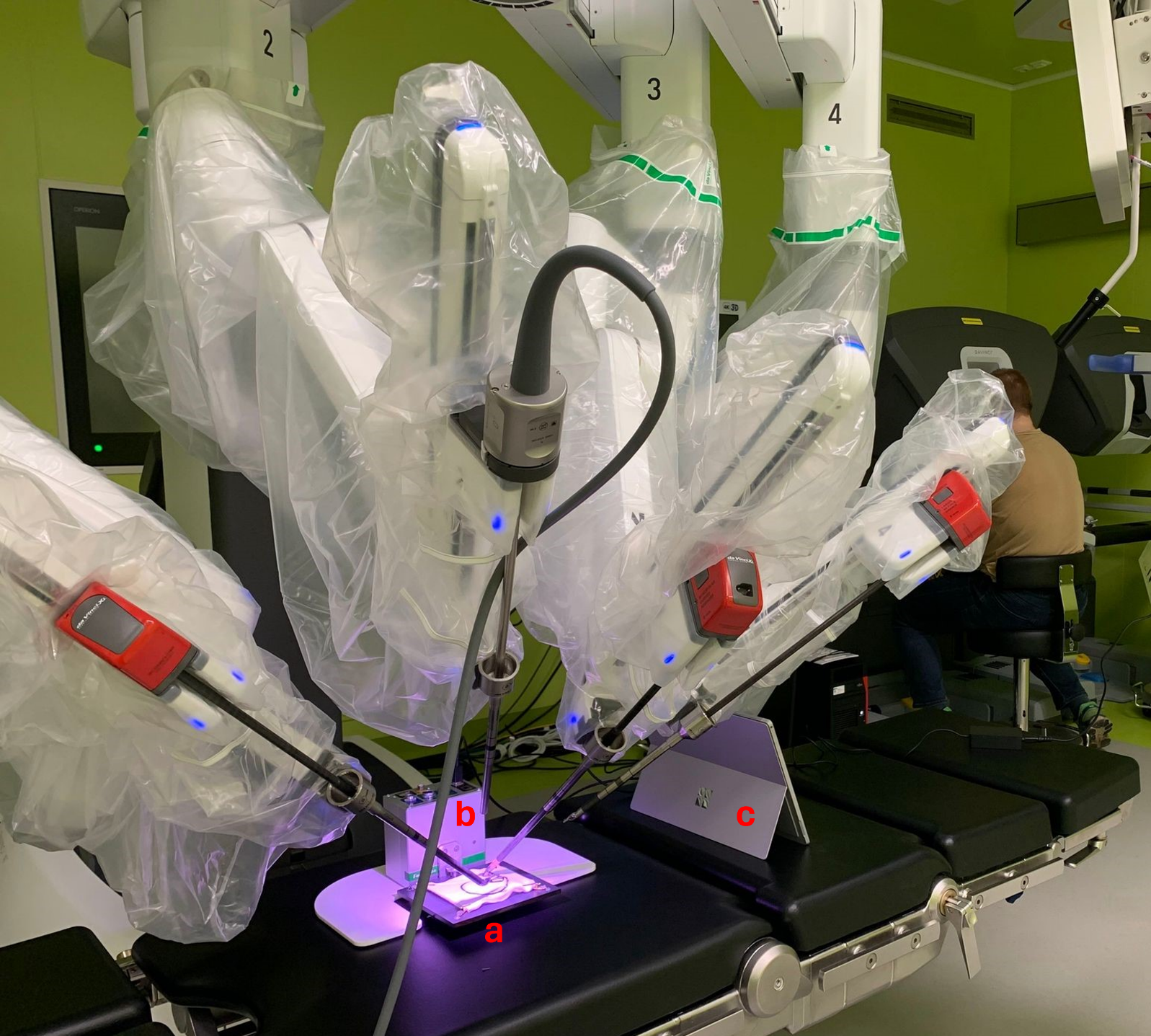


Supplementary Material Figure 1: The experimental setup measured the force interactions between DaVinci Xi robotic instruments and the task mounted on a platform (a) attached to the ForceTrap® sensor (b). Force inputs were analysed using the ForceSense software on a workstation (c).
